# Combination Therapy with Antibiotics and Anthrax Immune Globulin Intravenous (AIGIV) Is Potentially More Effective than Antibiotics Alone in Rabbit Model of Inhalational Anthrax

**DOI:** 10.1371/journal.pone.0106393

**Published:** 2014-09-16

**Authors:** Srinivas Kammanadiminti, Ravi Kumar Patnaikuni, Jason Comer, Gabriel Meister, Chris Sinclair, Shantha Kodihalli

**Affiliations:** 1 Department of Clinical Research, Cangene Corporation, Winnipeg, Manitoba, Canada; 2 Battelle Biomedical Research Center, West Jefferson, Columbus, Ohio, United States of America; Van Andel Research Institute, United States of America

## Abstract

**Background:**

We have evaluated the therapeutic efficacy of AIGIV when given in combination with levofloxacin and the effective window of treatment to assess the added benefit provided by AIGIV over standard antibiotic treatment alone in a New Zealand white rabbit model of inhalational anthrax.

**Methods:**

Rabbits were exposed to lethal dose of aerosolized spores of *Bacillus anthracis* (Ames strain) and treated intravenously with either placebo, (normal immune globulin intravenous, IGIV) or 15 U/kg of AIGIV, along with oral levofloxacin treatment at various time points (30–96 hours) after anthrax exposure.

**Results:**

The majority of treated animals (>88%) survived in both treatment groups when treatment was initiated within 60 hours of post-exposure. However, reduced survival of 55%, 33% and 25% was observed for placebo + levofloxacin group when the treatment was initiated at 72, 84 and 96 hours post-exposure, respectively. Conversely, a survival rate of 65%, 40% and 71% was observed in the AIGIV + levofloxacin treated groups at these time points.

**Conclusions:**

The combination of AIGIV with antibiotics provided an improvement in survival compared to levofloxacin treatment alone when treatment was delayed up to 96 hours post-anthrax exposure. Additionally, AIGIV treatment when given as an adjunct therapy at any of the time points tested did not interfere with the efficacy of levofloxacin.

## Introduction

*Bacillus anthracis*, the etiologic agent of anthrax, is a Gram-positive, spore-forming bacterium that can cause human disease when exposed via the gastrointestinal, cutaneous, or inhalation (pulmonary) routes with pulmonary exposure being the most lethal at close to 100% mortality in the absence of treatment [1]. The mortality caused by *B. anthracis* is predominantly due to three well characterized virulence factors; lethal factor (LF), edema factor (EF) and protective antigen (PA). Anthrax toxin includes lethal toxin (LT) and edema toxin (ET) which are binary complexes formed, by association between PA and LF or EF, respectively. Lethal toxin is the predominant cause of severe disease and death following inhalational spore exposure [2]. Vaccination is an effective pre-exposure prophylactic measure against anthrax disease. However, due to the rapid nature of the disease progression vaccination is unlikely to provide protection if given after an individual has been exposed to aerosolized spores or after the onset of clinical disease. Post-exposure prophylaxis therapy with antibiotics is indicated for inhalational anthrax.

Symptomatic anthrax patients are currently treated with antimicrobial agents with known activity against *B*. *anthracis*. Although these therapies can address bacteremia caused by the organism, they do not immediately inhibit circulating toxin, which plays a significant role in anthrax pathogenesis. Bactericidal effects can reduce the formation of new toxin through inhibition of bacteremia. In addition, antibiotic therapy for inhalational anthrax is generally effective if given within days of exposure. During the 2001 outbreak in which postal workers in US were exposed to anthrax spores, 5 of 11 (45%) patients treated with antibiotics after the onset of symptoms succumbed to disease during the course of treatment. This suggests that delayed antibiotic treatment is not 100% effective [3]–[5] and there is a considerable unmet need for antitoxin therapeutics targeting lethal and/or edema toxin to supplement traditional antibiotic therapy. To address this need, Cangene has developed a hyperimmune product; Anthrax Immune Globulin Intravenous (AIGIV) against the PA antigen of anthrax bacillus for licensure under the Animal Rule. The Animal Rule (21 CFR 601.90) provides an avenue for approval of products based on evidence of effectiveness derived from adequate and well-controlled animal studies when human trials are unethical or not feasible [6]. It is expected that novel anthrax therapeutics must demonstrate an additional benefit over the standard treatment with antibiotics. In this work, we report data from two independent placebo controlled studies in rabbits that evaluated the therapeutic efficacy of AIGIV compared to placebo (IGIV) when both were given in combination with levofloxacin at various time points after exposure to inhalation anthrax. The time points selected for intervention closely mimic the clinical scenario wherein patients are diagnosed with anthrax and receive appropriate treatments late in the disease progression. Results of these studies suggest that antibiotics on their own are highly efficacious when given at the onset of clinical disease and when they are delayed up to 60 hours post-exposure in the rabbit model of inhalational anthrax. However, an improvement in survival was observed with AIGIV+ levofloxacin compared to Placebo+ levofloxacin alone, when treatment was delayed up to 96 hours post-exposure.

## Materials and Methods

### Animals, Animal Husbandry and Veterinary Care

Specific pathogen free New Zealand white rabbits (*Oryctolagus cuniculus*), weighing 2.5 to 3.5 kg prior to study initiation, with surgically implanted vascular ports were obtained from Covance Laboratories (Denver, PA). Rabbits were housed individually in stainless steel cages on racks equipped with automatic watering systems. Rabbits were quarantined for at least 7 days prior to study initiation in accordance with test facility procedures. Following quarantine, rabbits that were in good health, free of malformations, and exhibited no signs of illness were placed on study. Each rabbit was acclimated to the jacket –tether system designed for infusion studies for a minimum of 3 days prior to infusion.

Animals were observed twice per day during the quarantine and pre-spore exposure period by trained personnel. Following exposure, animals were observed for clinical signs and mortality every 12 hours till the end of the study. Food consumption was monitored once per day. Any rabbit judged to be moribund by a trained technician, veterinarian, or by the study director, was euthanized immediately in accordance with Battelle's standard operating procedures. The pre-established criteria for euthanasia included: the presence of any seizure; severe respiratory distress; and unresponsive to touch or external stimuli. Rabbits that met criteria for euthanasia or at the end of the study were sedated with acepromazine (1–5 mg/kg subcutaneously) or other approved anesthetic and then were administered an overdose of a euthanasia agent containing pentobarbital or other American Veterinary Medical Association (AVMA) approved method of euthanasia.

### Ethics statement

The animal studies were conducted in a Biosafety Level 3 facility at Battelle Biomedical Research Center, West Jefferson, OH, USA. All studies were approved by the Battelle Biosafety Committee and the Institutional Animal Care and Use Committee (IACUC) of Battelle which adhere to the Animal Welfare Act and the Guide for the Care and Use of Laboratory Animals (Permit Number: A3034-01) [7], [8].

### Anthrax exposure

*Bacillus anthracis* (Ames strain) spores were used for aerosol exposure. A modified type three-jet Collison nebulizer (BGI, Waltham, MA) was used to generate a controlled delivery of aerosolized *B. anthracis* Ames spores from a liquid suspension into a muzzle-only exposure chamber. Rabbits were exposed to a targeted aerosol challenge dose of 200×LD_50_ [2.1×10^7^ spores] based on the established LD_50_ dose for rabbits [9]. The inhaled dose of anthrax spores for each animal was calculated as described previously [10], [11].

### AIGIV and placebo

AIGIV is a purified human IgG product manufactured using the plasma collected from healthy donors vaccinated with AVA (Anthrax Vaccine Adsorbed). It is a 5% solution with 59 mg/ml of total protein (>99% is human IgG) and a potency of 2.73 U/ml. The potency is measured by Toxin Neutralization Assay (TNA) using the dilution curve dose-response EC_50_ and the units are assigned based on an anti-AVA reference serum standard obtained from the Center for Disease Control (CDC). Placebo consists of normal human immune globulin; IGIV which is a 5% solution with 55 mg/ml of total protein manufactured using the plasma from normal individuals. Both AIGIV and placebo were manufactured using the similar process and supplied by Cangene Corporation, Winnipeg, Canada.

AIGIV or placebo was loaded into the infusion cassettes before infusion. A CADD-Legacy PLUS Model 6500 pump with a 50 ml cartridge was used to administer intravenous products. AIGIV and placebo were administered as a slow intravenous infusion (1.5 to 3.0 ml/kg/hour). A special jacket and tether system was used for infusion to avoid unnecessary restraint. AIGIV was administered at a dose level of 15 U/kg and the placebo was administered as a single dose with a volume of which was equivalent to that of AIGIV.

### Levofloxacin

Levaquin Oral Solution (levofloxacin 25 mg/ml, Ortho-McNeil-Janssen Pharmaceuticals) was administered as supplied at a dose of 50 mg/kg once daily for three consecutive days via oral gavage. The levofloxacin dose was chosen to closely mimic human pharmacokinetic parameters in rabbits.

### Bacteremia and toxemia

**Figure 1:**
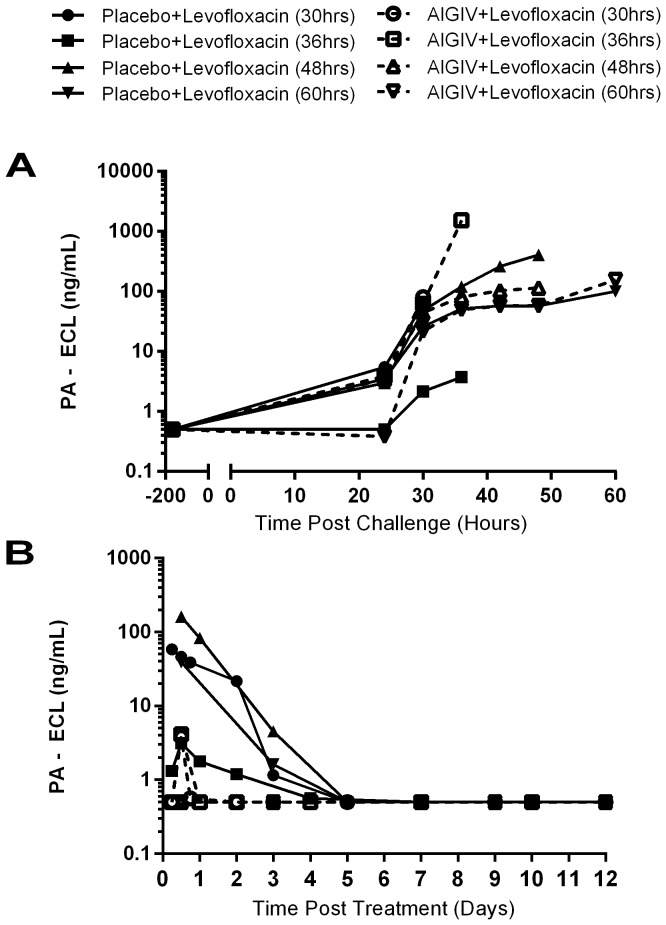
Pre-Treatment (Post-Challenge) and Post-Treatment Mean PA levels in Sera from Study 1 Rabbits. New Zealand white rabbits were exposed to 200×LD_50_ doses of aerosolized *B. anthracis* spores and serum collected at different time points Post-Challenge (A) and Post-Infusion (B) was tested by electro-chemiluminiscence (ECL) assay for detection and quantitation of *B. anthracis* protective antigen (PA). Animals received combination treatment with Placebo+ levofloxacin or AIGIV + levofloxacin at 30, 36, 48 and 60 hours post-exposure. AIGIV was given IV as a slow infusion at 15 U/kg of body weight and levofloxacin at 50 mg/kg given orally once a day for 3 days. PI = Post-Infusion PC = Post-Challenge.

Blood and serum samples were collected at various time points relative to spore exposure and treatment. The blood samples were collected at 6 (study 1) or 12 hours (study 2) intervals after anthrax exposure for analysis until treatment. In study 1, the serum samples were collected at six hour intervals from 24 to 48 hours and day 3, 5, 7, 9, 11, and 14 post-exposure. The post-treatment toxin data presented in Figure 1 was relative to time of treatment. The post- treatment samples for study 2 were collected at 1 hour, 12 hours, 24 hours, day 3, 5, 7, 10, 14, 21 and 32 (at termination) after the initiation of treatment.

The bacteremia was assessed by streaking whole blood on blood agar plates (Hardy Diagnostics, Santa Maria, CA, USA) and incubating at 37°C±2°C for up to 48 hours to determine the presence or absence of *B. anthracis* by macroscopic observation of bacterial colonies. The protective antigen (PA) in serum was quantitated by electrochemiluminescence (ECL) based assay using microplates coated with PA antibodies (Meso Scale Discovery System (MSD), Rockville, MD, USA). The bound PA is detected with the addition of the MSD detector antibody (STAG-labeled anti-PA antibody) using a MSD Sector Imager.

### Experimental study design

Two separate randomized placebo control studies were conducted. The treatment consisted of placebo+ levofloxacin and AIGIV+ levofloxacin. To minimize bias, rabbits were randomized by body weight to each of the treatment group for all studies. An equal number of male and female rabbits were included in each treatment group. Randomization was performed with SAS statistical software.

**Study 1.** Seventy-two specific pathogen free rabbits were randomized into nine treatment groups with 8 animals/group. Due to the large number of animals, the study was conducted in 3 sets of challenge days. Animals from each treatment time group were further randomized to the aerosol challenge days and on each challenge day, 24 rabbits were spore challenged with a minimum of 2 animals from each group. The challenge order within each challenge day was also randomized. The combination treatments were initiated at 30, 36, 48 or 60 hours post-spore exposure. At the indicated time points, levofloxacin was administered first orally and a single slow intravenous infusion with either AIGIV or placebo was initiated within 30 minutes. Two more doses of levofloxacin were administered at an interval of 24 hours. A total of 8 rabbits were exposed (2 or 3/each challenge day) to anthrax spores and left untreated as aerosol controls to confirm lethality of the spore exposure. Animals were monitored for survival for 30 days post-exposure.

**Study 2.** Two-hundred and forty-six specific pathogen free rabbits were randomized into four treatment times (60, 72, 84 and 96 hours). Treatment group size varied with the time of treatment due to anticipated pre-treatment mortality. A total of 18 rabbits were exposed (2/each challenge day) to anthrax spores and left untreated as aerosol controls to confirm lethality of the spore exposure. Due to the large number of animals, aerosol challenges occurred over 9 challenge days. The required number of animals from each treatment time group was further randomized to a challenge day. In the final randomization step, animals that were randomized to the challenge days were further randomized for the order of challenge. Rabbits that survived to the designated treatment time were randomly assigned to AIGIV+ levofloxacin and placebo+ levofloxacin treatment groups. Animals were monitored for survival for 32 days post-exposure.

All statistical analyses were conducted with Stata (Version 11.1) and all the tests were reported at the 0.05 level of significance. Two sided Fisher's exact tests were utilized to compare the survival rates between treatments and control group (untreated). Fisher exact test was also used to compare bacteremia and toxemia rates between treatment groups. The ANOVA models and Wilcoxon rank-sum tests were used for the toxemia level comparison.

The time-to-death data combined with survival data were analyzed. The Kaplan-Meier curves were plotted and the log-rank test was computed to determine if differences between groups were statistically significant. When the log-rank test was significant, pairwise log-rank tests were computed to determine which groups were different.

## Results

The most common clinical signs included anorexia, lethargy and respiratory distress. These observations were consistent with symptoms of inhalational anthrax.

In study 1, the average aerosol exposure dose for 72 animals was 178±40 *B. anthracis* LD_50_ equivalents. All untreated spore exposure control animals (8/8) succumbed to anthrax disease with a median time to death of 99.7 hours (Table 1A). None of the exposed animals in the placebo+ levofloxacin groups that received treatments starting at 30, 36, 48 and 60 hours post- exposure succumbed to disease except one animal from the 60 hours treatment group. Three exposed animals (one at 30 hours and two at 60 hours treatment group) treated with AIGIV+ levofloxacin died of anthrax infection.

**Table 1:** Summary of mean time to death and survival of rabbits in two sequential combination treatment studies.

	1A Study 1					
Treatment Time	Treatment	No. Exposed	No. Survived/No. Treated	Survival Rate^1^ (95% Confidence Interval)	Median Time to Death (hours)	p-value Pairwise Log- Rank Test^2^
Control	None	8	0/8	0.00 (0.00, 0.37)	99.72	-
30 hours	Placebo+Levofloxacin	8	8/8	1.00 (0.63, 1.00)	-	1.0000
	AIGIV+Levofloxacin	8	7/8	0.88 (0.47, 1.00)	-	
36 hours	Placebo+Levofloxacin	8	8/8	1.00 (0.63, 1.00)	-	NA
	AIGIV+Levofloxacin	8	7/7^3^	1.00 (0.59, 1.00)	-	
48 hours	Placebo+Levofloxacin	8	8/8	1.00 (0.63, 1.00)	-	NA
	AIGIV+Levofloxacin	8	8/8	1.00 (0.63, 1.00)	-	
60 hours	Placebo+Levofloxacin	8	7/8	0.88 (0.47, 1.00)	-	1.0000
	AIGIV+Levofloxacin	8	6/8	0.75 (0.35, 0.97)	-	

	1B Study 2					
Treatment Time	Treatment	No. Treated/No Exposed	No. Survived/No. Treated	Survival Rate^4^ (95% Confidence Interval)	Median Time to Death (hours)	p-value Fisher's Exact Test^5^
Control	None	0/18	0/18	0.00 (0.00, 0.19)	79.78	-
60 hours	Placebo+Levofloxacin	18/20	9/10	0.90 (0.55–1.00)	-	1.0000
	AIGIV+Levofloxacin		8/8	1.00 (0.63–1.00)	-	
72 hours	Placebo+Levofloxacin	43/72	11/20*	0.55 (0.32–0.77)	-	0.5450
	AIGIV+Levofloxacin		15/23	0.65 (0.43–0.84)	-	
84 hours	Placebo+Levofloxacin	19/64	3/9*	0.33 (0.07–0.70)	177.38	1.0000
	AIGIV+Levofloxacin		4/10	0.40 (0.12–0.74)	129.87	
96 hours	Placebo+Levofloxacin	15/72	2/8	0.25 (0.03–0.65)	127.05	0.1319
	AIGIV+Levofloxacin		5/7	0.71 (0.29–0.96)	-	

1 Survival rates for each group at day 30 post exposure.

2 Time-to-death and overall survival rates between groups by pairwise Log-rank test. NA- Log-rank test was not possible due to no deaths occurred in either group.

3 A total of 9 animals were exposed to anthrax spores but two were excluded from survival analysis due to death related to gavage error. - Not determined due to lack of sufficient number of deaths.

4 Survival rates for each group at day 32 post exposure.

5 Comparing overall-survival between the groups. *Two animals from 72 hours and one animal from 84 hours group were non bacteremic at the time of treatment.

There were no significant differences in the survival rates between the two treatment groups at any of the time points tested. Due to high survival rates in the levofloxacin group, any added benefit provided by AIGIV could not be demonstrated in this model. Therefore, a further study was necessary to identify a time point beyond 60 hours at which a reduced protection by human equivalent dose of levofloxacin is evident in order to evaluate the protective contribution of AIGIV when given in combination with levofloxacin.

In study 2, the average aerosol exposure dose for 246 animals was 282±71 *B. anthracis* LD_50_ equivalents. Anthrax exposed rabbits were treated at 60, 72, 84 or 96 hours post-exposure. A total of 228 animals were challenged with a target 200×LD50 *B. anthracis* spores. All exposed and untreated control animals in this study also succumbed to anthrax with a median time to death of 79.8 hours (Table 1B). Due to extreme delay in the treatment time, the majority (58%) of animals succumbed to anthrax infection before the treatment initiation. A delay in the treatment to 84 or 96 hours post-exposure resulted in a reduced survival rates (25–33%) for the placebo+ levofloxacin group compared to ∼100% survival when animals were treated before 60 hours post-exposure. A higher rate of survival was observed for AIGIV+ levofloxacin group compared to placebo+ levofloxacin (71% vs 25%) group when treatments were delayed to 96 hours post-exposure. These results did not achieve statistical significance (p = 0.139, Fisher's exact test) for the treatment effect due to the small sample size (n = 7 or 8) since most animals succumbed prior to treatment at 96 hours. Differences in survival at 72 and 84 hours trended higher for the AIGIV +levofloxacin group as compared with the placebo +levofloxacin, but were also not statistically significant. Table 1A and 1B summarizes the survival rates with confidence intervals for each group in studies 1 and 2, respectively.

### Bacteremia

In study 1, a total of 61/71(86%) anthrax exposed animals were bacteremic with a mean time to onset of 27.96 hours post-exposure (Table 2). The incidence of bacteremia prior to the treatment was 88% (7/8), 50% (4/8), 88% (7/8), and 100% (8/8) in the placebo+ levofloxacin groups treated at 30, 36, 48 and 60 hours, respectively. At these same time points, the incidence of bacteremia in AIGIV+ levofloxacin groups was 100% (8/8), 71% (5/7), 75% (6/8), and 100% (8/8), respectively. Following the administration of combination treatment, the bacteremia was resolved rapidly with no detectable bacteremia by 6–12 hours post-first treatment. In fact, only three animals were positive by culture at some point following treatment in all time points combined. All animals that were negative for bacteremia prior to treatment remained negative at all time points post-treatment. This confirmed the efficacy of levofloxacin in preventing the onset of bacteremia in exposed animals during this early treatment window as well as eliminating the bacteria efficiently in animals that were bacteremic at the time of treatment.

**Table 2:** Effect of combination treatment on incidence of bacteremia and toxemia in study 1.

Bacteremia				
Treatment Time	Treatment*	Time to Onset of Bacteremia (hours) Geometric Mean (Min, Max)	Bacteremia Incidence** No. Positive/No. Animals (%)	
			Pre-Treatment	Post-Treatment**
Control	None	29.49 (22.00.39.52)	8/8 (100%)	NA
30 hours	Placebo+Levofloxacin	26.11 (22.35, 31.55)	7/8 (88%)	0/8 (0%)
	AIGIV+Levofloxacin	27.36 (23.00,31.47)	8/8 (100%)	2/8 (25%)
36 hours	Placebo+Levofloxacin	28.99 (23.47, 34.40)	4/8 (50%)	0/8 (0%)
	AIGIV+Levofloxacin	23.94 (21.30, 27.47)	5/7 (71%)	0/7 (0%)
48 hours	Placebo+Levofloxacin	27.29 (22.18, 42.18)	7/8 (88%)	1/8 (13%)
	AIGIV+Levofloxacin	28.11 (25.02, 31.52)	6/8 (75%)	0/8 (0%)
60 hours	Placebo+Levofloxacin	29.57 (25.23,34.03)	8/8 (100%)	0/8 (0%)
	AIGIV+Levofloxacin	29.93 (23.93, 35.07)	8/8 (100%)	0/8 (0%)
Total		27.96 (21.30,42.18)	61/71 (86%)	3/63 (5%)
Placebo +Levofloxacin		-	26/32 (81%)	1/32 (3%)
AIGIV +Levofloxacin		-	27/31 (87%)	2/31 (6%)

Toxemia				
Treatment Time	Treatment*	Time to Onset of Toxemia(hours) Geometric Mean (Min, Max)	Toxemia Incidence** No. Positive/No. Animals (%)	
			Pre-Treatment	Post-Treatment
Control	None	30.04 (22.00, 45.72)	8/8 (100%)	NA
30 hours	Placebo +Levofloxacin	27.13 (22.35,31.55)	8/8 (100%)	8/8 (100%)
	AIGIV+Levofloxacin	26.62 (21.68, 31.47)	8/8 (100%)	2/8 (25%)
36 hours	Placebo +Levofloxacin	31.77 (27.98,34.40)	4/8 (50%)	4/8 (50%)
	AIGIV+Levofloxacin	23.94 (21.30, 27.47)	5/7 (71%)	2/7 (29%)
48 hours	Placebo +Levofloxacin	29.04 (22.18,42.18)	7/8 (88%)	7/8 (88%)
	AIGIV+Levofloxacin	27.18 (25.02,30.92)	7/8 (88%)	0/8 (0%)
60 hours	Placebo+Levofloxacin	29.57 (25.23,34.03)	8/8 (100%)	7/8 (88%)
	AIGIV+Levofloxacin	30.31 (22.82,45.53)	8/8 (100%)	0/8 (0%)
Total		28.33 (21.30,45.72)	63/71 (89%)	30/63 (48%)
Placebo+ Levofloxacin		-	27/32 (84%)	26/32 (81)
AIGIV + Levofloxacin		-	28/31 (90%)	4/31 (13%)

NA- not applicable as animals were not treated.** data from all time points up to treatment (pre-treatment) and all time points post-treatment were combined for overall incidence.

In study 2, a total of 92/95 (97%) animals were bacteremic prior to treatment with a mean time to first positive culture of 31.54 hours post-exposure (Table 3). The three animals that were negative for bacteremia prior to treatment remained negative to the end of the study. Only 4 of the 82 surviving rabbits were bacteremic one day after receiving the initial levofloxacin treatment and bacteremia was resolved in the majority of animals by day 5 post-treatment. There was no significant difference in the proportion of bacteremic animals between treatment groups at any of the treatment time point (p>0.05, Fisher's exact test). Together, the data from both treatment groups in these two studies demonstrate that rabbits treated with levofloxacin rapidly clear bacteria when administered with or without AIGIV (Table 3).

**Table 3:** Effect of combination treatment on incidence of bacteremia in study 2.

Treatment Time	Treatment*	Time to Onset of Bacteremia (hours)Geometric Mean (Min Max)^a^	Bacteremia Incidence (No. Positive/No. Animals (%))				
			Pre- Treatment	Post-Treatment**			
				24 hours	Day 3	Day 5	Terminal
60 hours	Placebo+ Levofloxacin	30.22 (23.37,36.57)	10/10 (100%)	1/9 (11.1%)	0/9 (0%)	0/9 (0%)	0/1 (0%)
	AIGIV +Levofloxacin	29.21 (23.60,49.55)	8/8 (100%)	0/8 (0%)^b^	0/8 (0%) b	1/8 (13%) b	—
72 hours	Placebo+ Levofloxacin	27.87 (20.48,49.13)	18/20 (90%)	0/15 (0%)	0/12 (0%)	1/11 (9%)	0/9 (0%)
	AIGIV +Levofloxacin	32.13 (21.20,73.27)	23/23 (100%)	0/22 (0%) b	0/19 (0%) b	0/16 (0%) b	0/8 (0%) b
84 hours	Placebo+ Levofloxacin	29.55 (21.20,37.70)	8/9 (88.9%)	0/8 (0%)	0/5 (0%)	0/3 (0%)	1/6 (17%)
	AIGIV +Levofloxacin	35.77 (21.40.78.58)	10/10 (100%)	1/9 (11.1%) b	0/5 (0%) b	0/4 (0%) b	0/5 (0%) b
96 hours	Placebo+ Levofloxacin	38.38 (22.32,72.53)	8/8 (100%)	1/5 (20%)	0/3 (0%)	0/2 (0%)	1/5 (20%)
	AIGIV +Levofloxacin	37.88 (19.50,85.37)	7/7 (100%)	1/6 (16.7%) b	0/5 (0%) b	0/5 (0%) b	0/2 (0%) b

a The overall mean onset time for all groups is 31.54 (19.50, 85.37).

b Pairwise comparison Fisher's exact test p>0.05 AIGIV+levofloxacin vs. placebo+levofloxacin.

— No animal died.

**Post-treatment blood collection was relative to the end of infusion (after the first levofloxacin dose).

### Toxemia

In study 1, 63/71 (89%) anthrax exposed animals exhibited toxemia (as measured by detectable levels of PA using an ECL assay) with a mean onset time of 28.33 hours post-exposure (Figure 1A, Table 2). Following treatment, a resolution of toxemia was observed in majority (87%) of the AIGIV+ levofloxacin treated animals by 24 hours post-treatment (Figure 1B). However, the persistence of PA in circulation was observed in placebo+ levofloxacin groups up to 5 days following treatment (Figure 1B). Eighty-one percent of animals (26/32) treated with placebo + levofloxacin exhibited detectable circulating PA (limit of detection is 1 ng/ml), while only 13% (4/31) animals treated with AIGIV+ levofloxacin exhibited PA levels post-treatment (Table 2).

Similarly, in study 2, rabbits became toxemic as early as 20 hours post-exposure (first blood draw after aerosol exposure) with a mean time to toxemia of 29.42 hours (Table 4).

**Table 4:** Effect of combination treatment on incidence of toxemia in study 2.

Treatment Time	Treatment*	Time to Onset of Toxemia (hours)^a^ Geometric Mean (Min, Max)	Toxemia (No. Positive/No. Animals (%))						
			Pre- Treatment	Post -Treatment**					
				1 hour	12 hours	24 hours	Day 3	Day 5	Terminal
60 hours	Placebo + Levofloxacin	29.03 (23.37, 36.57)	10/10 (100%)	10/10 (100%)	9/9 (100%)	9/9 (100%)	7/9 (78%)	0/9 (0%)	1/1 (100%)
	AIGIV + Levofloxacin	31.17 (23.60, 37.20)	8/8 (100%)	0/8 (0%) b	0/8 (0%) b	0/8 (0%) b	1/8 (13%) b	0/8 (0%)	—
72 hours	Placebo + Levofloxacin	28.42 (20.48, 61.45)	19/20 (95%)	18/20 (90%)	13/16 (81%)	12/15 (80%)	5/12 (42%)	0/11 (0%)	8/9 (89%)
	AIGIV + Levofloxacin	29.67 (19.95,70.83)	23/23 (100%)	2/23 (9%) b	1/23 (4%) b	0/22 (0%) b	0/19 (0%)	0/16 (0%)	1/8 (13%)
84 hours	Placebo + Levofloxacin	28.79 (21.20, 37.70)	9/9 (100%)	9/9 (100%)	8/8 (100%)	8/8 (100%)	0/5 (0%)	0/3 (0%)	4/5 (80%)
	AIGIV + Levofloxacin	30.09 ( 21.40, 38.05)	10/10 (100%)	2/10 (20%) b	0/9 (0%) b	2/9 (22%) b	0/5 (0%)	0/4 (0%)	2/4 (50%)
96 hours	Placebo+ Levofloxacin	32.77 (22.32,37.13)	8/8 (100%)	8/8 (100%)	8/8 (100%)	5/5 (100%)	2/3 (67%)	0/2 (0%)	5/5 (100%)
	AIGIV + Levofloxacin	30.21 (19.50,48.90)	7/7 (100%)	0/7 (0%) b	0/6 (0%) b	1/6 (17%) b	0/5 (0%)	0/5 (0%)	1/2 (50%)

a The overall mean time to onset was 29.42 ( 19.50, 70.83).

— No animal died. ** Blood collections for post-treatment started 1 hour after infusion with AIGIV and one dose of levofloxacin.

b Fisher's exact test, p<0.05 IGIV+levofloxacin vs. placebo+levofloxacin.

**Figure 2:**
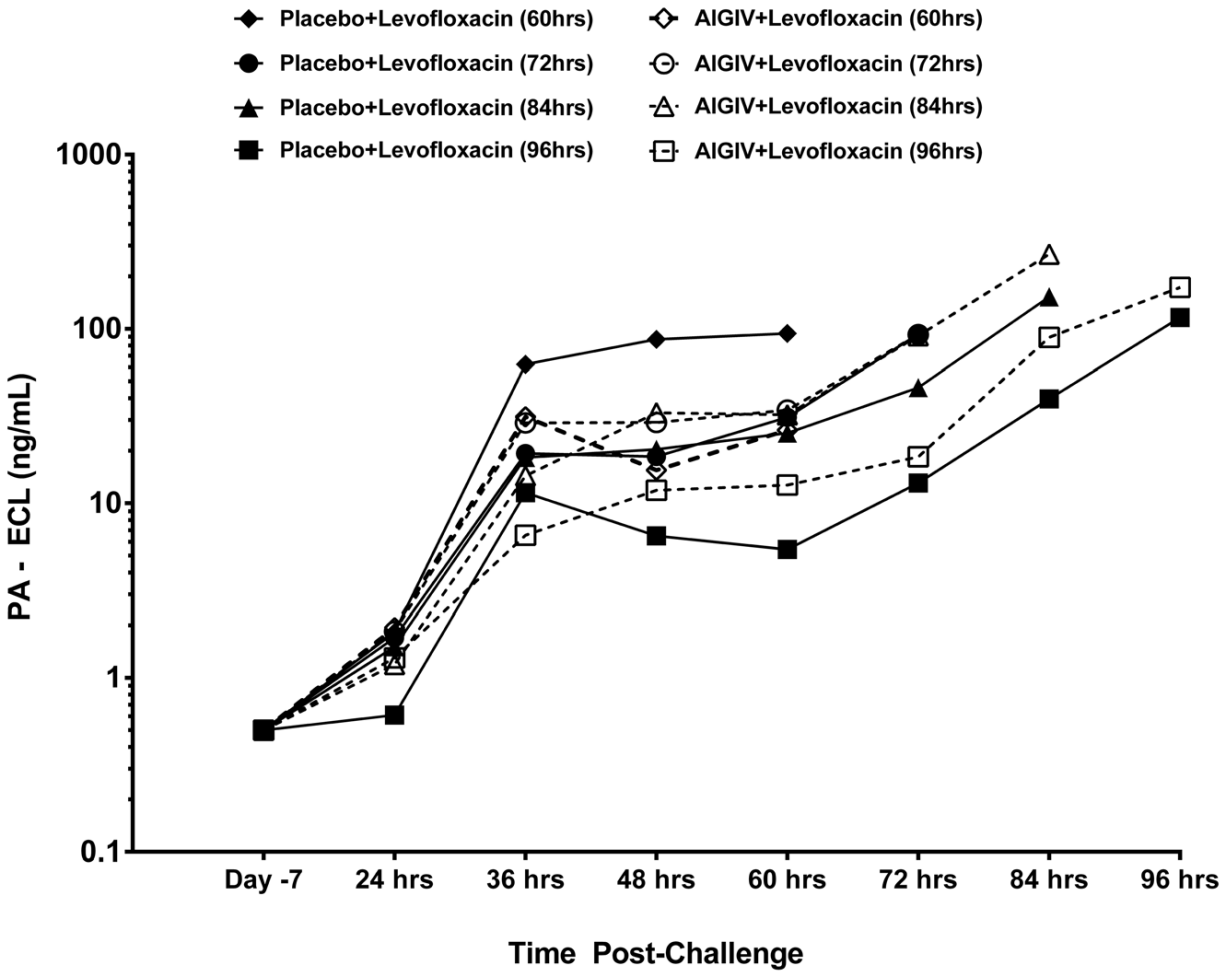
Pre-Treatment Mean PA levels in Sera from Study 2 Rabbits. New Zealand white rabbits were exposed to 200×LD_50_ doses of aerosolized *B. anthracis* spores and serum collected at different time points was tested by electro-chemiluminiscence (ECL) assay for detection and quantitation of *B. anthracis* protective antigen (PA). PI = Post-Infusion PC = Post-Challenge.

**Figure 3:**
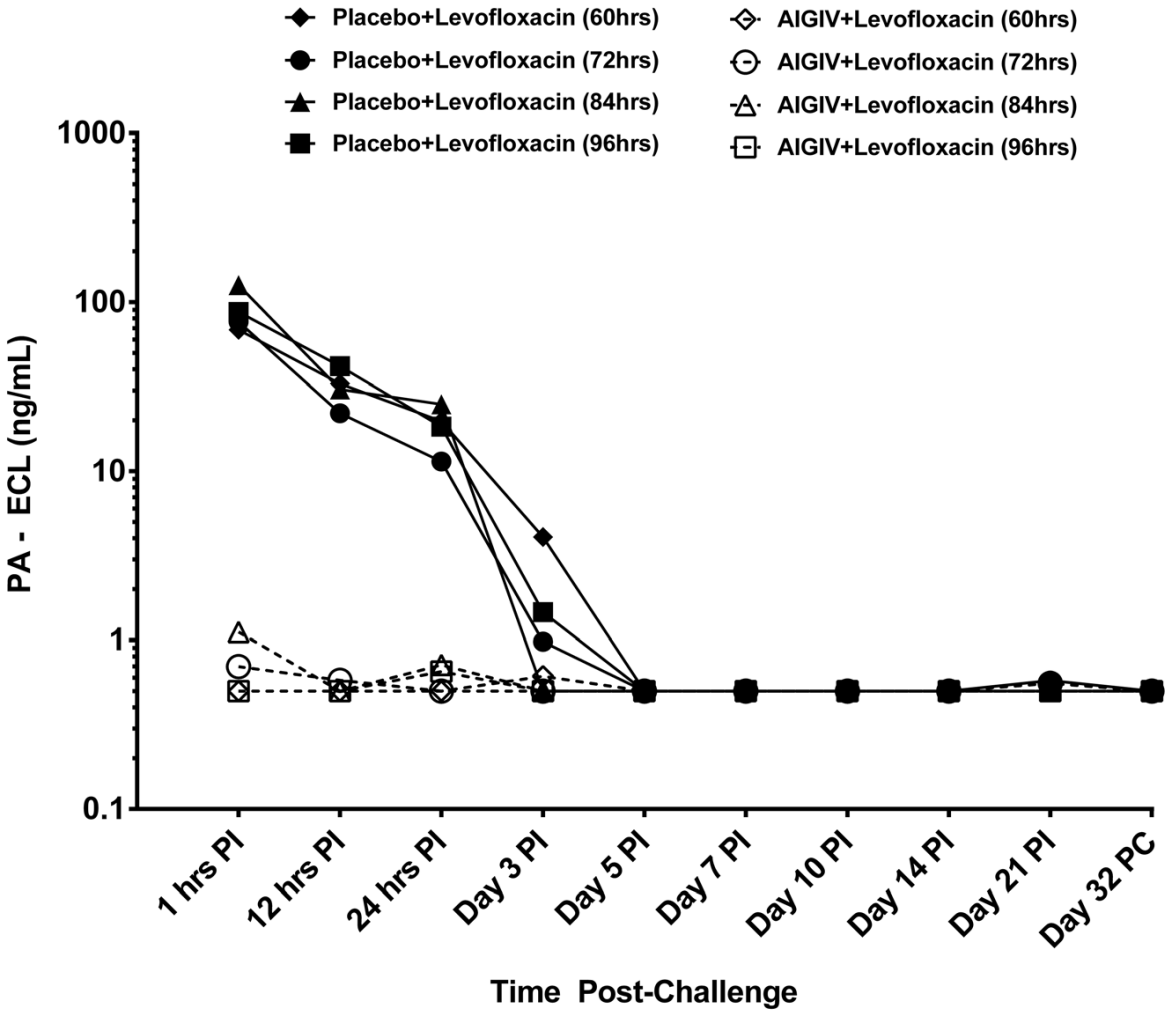
Post-Treatment Mean PA levels in Sera from Study 2 Rabbits. Animals received combination treatment with Placebo+ levofloxacin or AIGIV + levofloxacin at 60, 72, 84 and 96 hour post-exposure. AIGIV was given IV as a slow infusion at 15 U/kg of body weight and levofloxacin at 50 mg/kg given orally once a day for 3 days. The levels of PA were significantly reduced (P<0.05; ANOVA model and Wilcoxon rank sum test) following treatment with AIGIV and levofloxacin compared to treatment with IGIV and levofloxacin. PI = Post-Infusion PC = Post-Challenge.

There was a significant decrease in the proportion of rabbits with toxemia in AIGIV+ levofloxacin groups (3/45) compared to placebo+ levofloxacin (34/37) groups based on data at 24 hours post- treatment (p<0.05, Fisher's exact test). The greatest difference in the treatment efficacy between the treatment groups became apparent when the levels of circulating PA were compared (Figure 2 and Figure 3). The levels of PA were significantly reduced (p<0.05; ANOVA model and Wilcoxon rank sum) within 1 hour following treatment with AIGIV+ levofloxacin compared to gradual decline in PA levels for the placebo+ levofloxacin group over 3 days. This suggests that in the AIGIV +levofloxacin group, the AIGIV was able to clear circulating PA very rapidly while in the placebo+ levofloxacin group; there was a gradual decline of PA attributed to the resolution of the bacteremia.

## Discussion

Efficacy of antibiotics when given as post-exposure prophylaxis (PEP) has been well established in non human primate model of inhalation anthrax and these studies have formed the basis for FDA's approval of these antibiotics for post-exposure prophylactic treatment of inhalation anthrax [12]–[16]. However, there is limited data available on the efficacy of the antibiotics in animal models when administered after the onset of symptoms [17], [18], [19], [20]. In the US 2001 anthrax letter attack, a fatality rate of 50% was observed in humans even with the antibiotic treatment. Antibiotic treatment for inhalational anthrax is effective if started immediately after exposure but may be less effective if delayed even by hours [2]. Any added benefit with the use of AIGIV over levofloxacin alone has important implications in the management of exposed individuals given the highly lethal nature of inhalational anthrax infections.

The intent of the current investigation was to determine whether antitoxin therapy would provide beneficial effect in terms of survival over that of placebo when treatments are administered in conjunction with levofloxacin in a well established model of inhalational anthrax. We chose a human equivalent dose of levofloxacin in rabbits that closely mimic human pharmacokinetic parameters and treatment delays from 30–96 hours [20].

In our first study, a combination treatment of AIGIV with levofloxacin was initiated in rabbits at 30, 36, 48 or 60 hours post-exposure to lethal doses of aerosolized spores of *B. anthracis.* The starting time point of 30 hours was selected based on the preliminary model development studies and published data which suggested the onset of clinical disease at approximately 30 hours post- exposure [10]. The three delayed time points were selected as these mimic the clinical scenario wherein administration of any therapy is most likely delayed in patients exhibiting symptomatic anthrax due to late recognition of the disease after the onset of symptoms and/or due to the delay in distribution of medical countermeasures in an emergency. It is hypothesized that by delaying the treatments after the onset of clinical disease, the efficacy of antibiotic could be reduced thereby giving an opportunity for demonstration of added benefit of adjunct therapy with AIGIV.

These results demonstrate that AIGIV treatment does not interfere with the protective benefit observed with levofloxacin in animals suffering from inhalational anthrax. However, there was no significant difference in the survival rates observed between levofloxacin + placebo or levofloxacin +AIGIV treatment groups even when the treatment was delayed up to 60 hours post-exposure suggesting that a breakthrough in the survival provided by the antibiotics is beyond 60 hours post-exposure. This is not surprising as the available animal data suggest that bacterial replication can be controlled even at a high spore challenge dose with standard antibiotics alone [19], [20]. Recently, the administration of fully humanized monoclonal antibody directed against PA along with levofloxacin or ciprofloxacin was shown to be effective in preventing death of exposed rabbits and monkeys (>85%) when given at the onset of the clinical disease but did not demonstrate added benefit [20]. However, when the treatment initiation was delayed to 84 hours, a trend in added benefit by the monoclonal anti-PA antibody over levofloxacin alone was demonstrated [21].

The second set of AIGIV experiments investigated even longer delays in treatment in order to determine if there is a treatment time at which levofloxacin treatment results in a moderate or low proportion of survival. The goal was to establish a partially protective levofloxacin treatment regimen so that the ability of AIGIV to augment recovery in cases of symptomatic inhalational anthrax could be assessed. Consequently, the treatment was delayed beyond 60 hours to achieve a reduction in antibiotic efficacy. Our data from this study indicate that long delays in treatment (beyond 60 hours) result in the desired population of rabbits that cannot be completely saved by levofloxacin alone, but the delay results in a majority (58%) of the animals not surviving until treatment is administered.

The majority of animals were bacteremic (97%) and toxemic (99%) prior to treatment, indicating that most rabbits were treated in a therapeutic manner. While there was enhanced survival in the groups treated with AIGIV and levofloxacin compared to those treated only with levofloxacin at all of the intervention time points, a substantial degree of added benefit with AIGIV+ levofloxacin (71%) compared to those receiving placebo+ levofloxacin (25%) was observed when treatment was delayed to 96 hours post-exposure. An overall improvement in the survival rate of 46% was observed in AIGIV+ levofloxacin group compared to the placebo + levofloxacin group at 96 hours. However, the number of animals that survived to treatment was small due to the high rate of mortality observed prior to treatment. Although larger studies are needed to achieve the statistical significance of the added benefit provided by AIGIV above that of levofloxacin at 96 hours post- anthrax exposure, the key factor for demonstrating added benefit appears to be a time for delay of antibiotic treatment at which there is a reduction in survival benefit provided by antibiotics alone, likely due to the level of toxemia that is not directly impacted by the antibiotics.

The requirement for a prolonged course of antimicrobials as post-exposure prophylaxis to prevent anthrax that results from the germination of retained spores is well established [12]–[15], [22], [23]. The results of our current study suggest that a prolonged course of levofloxacin is not necessary to treat the established disease when combined with antitoxins. These results are in agreement with the recent findings by Vietri et al., 2009 [17] where a short course of antibiotic was protective when administered after the onset of bacteremia in a non-human primate model of inhalation anthrax.

In both AIGIV studies, there was an immediate and efficient reduction in PA concentration in groups treated with a single infusion of AIGIV compared to placebo when both were given with antibiotics. This data suggests an additional benefit of AIGIV therapy in combination with levofloxacin as this approach addresses the toxin mediated aspect of anthrax disease, consistent with the proposed indication of AIGIV for the treatment of toxemia associated with inhalational anthrax.

A large observed added benefit for antibody product treatment with antimicrobials over antimicrobial alone treatment has been reported in animal models [20], [24], [25]. On the other hand, some polyclonal PA antibodies given as combination therapy along with ciprofloxacin at the onset of clinical disease with high levels of circulating bacteremia/toxemia failed to demonstrate complete benefit [18]. This might be a consequence of low levels of the antibody circulatory concentration or severe damage to the animals as a result of the high bacteremia and toxemia concentrations prior to initiation of treatment [18]. In our study, there was a trend suggesting added benefit of AIGIV in terms of survival when the exposure dose was very high (200×LD_50_) and the treatment was delayed to 96 hours after exposure.

In summary, AIGIV has shown a substantial, but not statistically significant, added benefit when administered as an adjunct therapy with a short course of levofloxacin in a rabbit model of inhalation anthrax. These results suggest that a prolonged course of antibiotics is not necessary to treat the established disease from inhalational anthrax when combined with antitoxins. We have determined that there is a clinically meaningful trend toward improved survival with AIGIV+ levofloxacin over levofloxacin alone in the later treatment window when a delay in treatment time after exposure to anthrax. Unfortunately, this delay in treatment results in high pre-treatment mortality. Due to this high pre-treatment mortality large animal numbers are needed to demonstrate the significance of this effect.
